# Present and Future in the Treatment of Diabetic Kidney Disease

**DOI:** 10.1155/2015/801348

**Published:** 2015-04-07

**Authors:** Borja Quiroga, David Arroyo, Gabriel de Arriba

**Affiliations:** ^1^Nephrology Unit, Hospital Universitario de Guadalajara, Spain; ^2^Nephrology Unit, Hospital Universitari Arnau de Vilanova, Lleida, Spain; ^3^Medicine and Medicine Specialities Department, Universidad de Alcalá (UAH), Madrid, Spain

## Abstract

Diabetic kidney disease is the leading cause of end-stage renal disease. Albuminuria is recognized as the most important prognostic factor for chronic kidney disease progression. For this reason, blockade of renin-angiotensin system remains the main recommended strategy, with either angiotensin converting enzyme inhibitors or angiotensin II receptor blockers. However, other antiproteinuric treatments have begun to be studied, such as direct renin inhibitors or aldosterone blockers. Beyond antiproteinuric treatments, other drugs such as pentoxifylline or bardoxolone have yielded conflicting results. Finally, alternative pathogenic pathways are being explored, and emerging therapies including antifibrotic agents, endothelin receptor antagonists, or transcription factors show promising results. The aim of this review is to explain the advances in newer agents to treat diabetic kidney disease, along with the background of the renin-angiotensin system blockade.

## 1. Introduction

Diabetes mellitus (DM) and chronic kidney disease (CKD) have become two of the fastest growing pathologies worldwide [1, 2], while diabetic kidney disease (DKD) is still the leading cause of CKD and end-stage renal disease [2]. Population ageing and increase in prevalence of many interrelated comorbidities suggest that these numbers will worsen in the near future [3].

Despite emerging strategies and constant investigation, no current single treatment has been able to reverse or at least stop DKD progression. At best, some of the measures can partially slow the speed at which renal function is lost.

There are several possible reasons for this fact. First, most clinical trials have been addressed to evaluate the effect on albuminuria. Although albuminuria probably remains as the most influencing prognostic factor, up to one-fourth of normoalbuminuric diabetic patients will eventually develop CKD [4–6]. This has raised questions about the suitability of albuminuria as a surrogate marker in clinical trials, and renal function decline still remains as the most important target of nephroprotection [7, 8]. On the other hand, a growing body of evidence is uncovering various mechanisms of renal injury in the context of DM, leading to the appearance of potential novel drugs.

In this review, we summarize the available evidence regarding classical treatments for diabetic nephropathy, as well as novel agents, paths, and targets under basic and clinical investigation.

## 2. The Classical Nonspecific Measures

### 2.1. Glycemic Control

DKD occurs in approximately 20% of diabetic patients, and it can appear despite a good glycemic control [9]. Nevertheless, many important studies have demonstrated that a tighter glycemic control can delay the onset of DKD and slow its progression, beyond its well-known cardioprotective effect. This effect has been proved valid in both type 1 and type 2 DKD and in the short and long terms [10–16]. However, the risk of severe hypoglycemic adverse events prompted a change in international guidelines, which currently recommend individualization in treatment intensity according to patients' characteristics [17, 18]. Glycemic control can be achieved through diverse pharmacological treatments. Some of them, such as incretin degradation inhibitors or glucagon-like peptide analogues, may have specific nephroprotective effects independent of their glycemic impact, but these results require confirmation [19, 20].

### 2.2. Blood Pressure Control

Given the pathogenetic importance of intraglomerular hypertension in the initiation of DKD, earlier guidelines recommended a stricter blood pressure control in diabetic patients [21]. The latest 2012 KDIGO guidelines maintain a tighter blood pressure recommendation for proteinuric patients, regardless of etiology [22]. However, more recent data from several studies in the field of hypertension have evidenced the risks of hypotensive episodes and their vascular consequences [23, 24]. Hence, similarly to the evolution of recommendations in glycemic control, a more individual approach to blood pressure targets is advised [17].

### 2.3. Weight Loss

Overweight and obesity are frequent comorbidities to diabetes and play an important role in the pathogenesis of CKD [25]. This may be due both to a further increase in hyperfiltration and to specific hormonal dysregulations related to adipokines [26]. Weight loss in obese diabetic patients has been shown to markedly reduce albuminuria [27]. A decrease in serum creatinine has also been demonstrated in very hypocaloric diets, but this effect could be secondary to muscular mass loss [28]. There is also growing evidence about the beneficial effects of bariatric surgery in morbid obese patients over diabetes, renal function, and albuminuria [29, 30], but no trial has been yet specifically designed to analyze this effect on DKD.

### 2.4. Protein Restriction

Dietary advice in DKD patients is a complex issue: it compels carbohydrate consumption regulation, but the frequent concurrence of comorbidities also requires a low-salt diet for hypertension, fat-free for dyslipidemia, and hypocaloric intake for obesity. There is evidence of the benefits of moderate protein restriction up to 0.8 g/kg/day [31–33], and this indication is included in international guidelines at least for patients with reduced glomerular filtration rates (GFR) [21].

### 2.5. Smoking Cessation

Cigarette smoking has been linked to the appearance and progression of DKD, probably due to oxidative stress stimulation, and the cessation of this habit has also been associated with slower progression of the nephropathy [34–36]. If not for this reason, strong smoking cessation support should be offered to all diabetic and/or CKD patients as a means to reduce their increased vascular risk.

## 3. Past and Present: Renin-Angiotensin-Aldosterone System Blockade

### 3.1. ACEI and ARB

One of the most important risk factors for kidney disease progression in diabetic patients is the onset and persistence of proteinuria [37]. The use of angiotensin converting enzyme inhibitors (ACEI) or angiotensin II receptor blockers (ARB) to reduce proteinuria is currently the first-step strategy [17, 18, 38]. This benefit is valid for both type 1 and type 2 diabetic patients, even with low-grade proteinuria and normal GFR [39, 40].

Many clinical trials have been performed in this respect. A different approach that has attracted much attention has aimed to demonstrate the usefulness of combining two or even three of these drugs. The efficacy for lowering proteinuria with the combination of renin-angiotensin-aldosterone system blockers is at least the same as using one of them at maximum dosage. However, published studies have not succeeded in demonstrating these positive outcomes with the same adverse effects (renal function decline or hyperkalemia in the combination arms of several trials forced to stop some of them). While using ARB or ACEI to lower proteinuria in DKD and in proteinuric CKD is considered mandatory (evidence grade 1A), the lack of positive studies has encouraged a change in current recommendations against the use of dual blockade [41–43]. In spite of this fact, dual blockade is spreading more than ever, as shown in a recent retrospective study that included a great number of diabetic patients [44].

In a more detailed analysis of these trials (Table 1), ACEI and ARB combined treatment efficiently decreases proteinuria, and adverse events are usually limited to hyperkalemia and renal impairment [44]. For these reasons, we recommend that, in adequately selected cases with very high urinary protein excretion, dual blockade can probably be tried as long as a close monitoring can be ensured [50].

The unsolved issue is probably to find the optimal drug doses. The ROAD study showed that uptitration to the highest tolerated dose can be an interesting strategy to avoid adverse effects while achieving the maximum reduction in proteinuria [51]. In this sense, the use of a combination with equipotent doses of ACEI and ARB is not supported, due to a lack of benefits in terms of proteinuria as shown in the PRONEDI trial [42].

The controversy about an early treatment of nonproteinuric diabetic patients still remains. In the ROADMAP trial, proteinuria had a delayed onset in those patients treated with olmesartan, although at the expense of higher rates of cardiovascular events [23]. This benefit in primary prevention of DKD had been previously demonstrated with trandolapril in the BENEDICT trial [52]. A review and meta-analysis of the Cochrane Database concluded that ACEI could reduce the risk for new onset of albuminuria, but this effect cannot be proved with the use of ARB [53].

### 3.2. Aliskiren

Another option that has been proposed is the use of a selective inhibitor of human renin in combination with an ACEI, an ARB, or an aldosterone blocker. Aliskiren is a direct renin inhibitor that has been tested as an antiproteinuric agent in DKD. The AVOID trial generated important evidence about the efficacy of this drug with a nonsignificant rise of adverse effects (the aliskiren group developed more hyperkalemia, but the difference did not achieve statistical significance). However, the security profile of this treatment was questioned after the premature stop of the ALTITUDE trial due to a higher rate of adverse effects in an intermediate analysis [45, 46]. For this reason, the use of aliskiren in combination with ACEI/ARB is not supported for lowering proteinuria in kidney disease.

### 3.3. Spironolactone and Eplerenone

The benefits of the addition of an aldosterone blocker to the standard therapy in DKD have been noted in some clinical trials [54, 55]. Beyond the mere addition of spironolactone or eplerenone, these drugs have demonstrated slight renoprotective superiority in small studies compared to ACEI or ARB therapies [56]. For example, a recent study conducted by Esteghamati et al. including 136 patients that were using dual blockade with ACEI and ARB demonstrated that the substitution of the first one by spironolactone provides additional benefits in terms of proteinuria reduction with the same profile of adverse effects after 18 months of follow-up [48]. However, a reduction in GFR was noted in the spironolactone group, independent of blood pressure control. In CKD, this drop in renal function has been reported by other authors, but it appears reversible after the first treatment weeks [57, 58]. Although this beneficial effect is not well understood, it is hypothesized that these drugs avoid the aldosterone escape that happens in up to 40% of patients treated with an ACEI (Table 1) [47]. In an interesting study conducted by Sato et al., 55 patients received maximum doses of an ACEI. Of these, 18 patients showed aldosterone escape, so spironolactone was started. After 24 weeks, proteinuria was significantly reduced, showing no adverse effects [57].

Regarding the use of eplerenone, only one clinical trial has assessed the antiproteinuric value of this aldosterone blocker [49]. Epstein et al. demonstrated in a randomized double-blind study that eplerenone decreased albuminuria in diabetic patients at 4-, 8-, and 12-week follow-up. No differences in adverse effects were seen.

In spite of these good results, and given that these trials were only performed in early CKD stages, we must still be cautious until larger studies with long-term follow-up are published. Potential adverse events must still be closely monitored, especially hypotension, hyperkalemia, and renal failure [59].

## 4. Present: Beyond Renin-Angiotensin-Aldosterone System

Blocking renin-angiotensin system is not always enough to avoid proteinuria, so other approaches have been proposed. The formerly unexplored fields of inflammation and oxidative stress now become more important as targets for new treatments. Unfortunately, most of the studies performed yield incomplete conclusions or results that have not been confirmed in other studies [60].

### 4.1. Bardoxolone Methyl

Bardoxolone methyl is an antioxidant agent that activates Keap1-Nfr2 [nuclear 1 factor (erythroid-derived 2)-related factor 2] pathway and regulates inflammation in the kidney. However, the inhibition of this pathway focusing on slowing CKD progression in diabetic animals has produced controversial conclusions [61, 62]. Nrf2-deficient mice do not develop hyperfiltration in response to hyperglycaemia but experience a faster decline in renal function. In addition, some studies report different degrees of proteinuria in diabetic Nrf2 knock-out mice.

In humans, two relevant clinical trials have been published with bardoxolone methyl in DKD. The BEAM trial included 227 CKD patients (GFR between 20 and 45 mL/min/1.73 m^2^) that were randomized to placebo or various doses of bardoxolone for 52 weeks [63]. GFR increased significantly in all the bardoxolone arms, with a peak at 12 weeks that then remained stable. One of the most important results of the study regarding renal function was that albumin-to-creatinine ratio (ACR) was raised inversely to GFR in the treatment group. However, four weeks after treatment discontinuation, ACR returned to baseline levels. Besides, adverse effects were more frequent in the bardoxolone groups, especially muscle spasms (that reached 61% in the 75 mg group).

The BEACON trial was designed to confirm the findings of the BEAM trial, but it was stopped prematurely due to unacceptable high rates of cardiovascular events in the bardoxolone methyl arm [64]. In this study, 2185 type 2 diabetic CKD patients were randomized to receive placebo or 20 mg/day of bardoxolone methyl. A composite cardiovascular endpoint (nonfatal myocardial infarction, stroke, heart failure, or cardiovascular death) was achieved after a median exposure time to the drug of 7 months, so the trial was terminated due to safety concerns.

Recently, a derivative of bardoxolone methyl, an Nrf2 agonist called* dh404*, has shown beneficial effects in mice via decreasing inflammation and oxidative stress, but only at low doses. This study reopens the interests on the Nrf2 pathway in renoprotection in DKD [65].

### 4.2. Vitamin D Receptor Activation: Paricalcitol

Vitamin D is a well-known modulator of many different processes, and its deficiency can drive abnormalities in immune system, inflammation, or even cardiovascular events [49]. In addition, lower 25-OH-vitamin D levels have been independently linked to DKD progression in a subanalysis of the PRONEDI study [66]. The pleiotropic effects of vitamin D receptor activation have aroused a growing interest in some drugs, such as paricalcitol [67].

The presence of vitamin D receptors in podocytes has promoted several clinical studies, with the hypothesis of an effect of podocyte modulation on proteinuria. Agarwal et al. designed a small trial of 113 diabetic patients randomized to placebo or paricalcitol, demonstrating proteinuria reduction with paricalcitol qualitatively assessed by dipstick [68]. This effect was later confirmed in the VITAL study, published by de Zeeuw et al. [69]. In this study, 281 patients were randomized to receive placebo or 1 or 2 *μ*g/day of paricalcitol for 24 weeks. Only 40% of the patients were receiving maximum doses of ARB or ACEI, and the median of urinary ACR was 612.3 mg/g [70]. Proteinuria was not reduced in patients with paricalcitol at any dose when compared to placebo, but albuminuria was significantly reduced in patients with higher sodium intakes. It should be noted that only 58% of the patients assigned to 2 *μ*g/day of paricalcitol received the full dose during the whole study, due to adverse effects.

A recent paper published by Eren et al. demonstrates that the combination of paricalcitol with other drugs such as aliskiren can reduce DKD progression in rats beyond the simple reduction of proteinuria, when the renin-angiotensin system is adequately blocked [71]. In this study, the main finding was that paricalcitol in association with aliskiren reduced interstitial fibrosis.

A recent systematic review that included clinical trials about the effect of active vitamin D (both paricalcitol and calcitriol) on the control of proteinuria in CKD concludes that these drugs provide a significant reduction in proteinuria in addition to rennin-angiotensin system blockade. However, except the VITAL trial, the rest of the included studies were small in sample size, and the underlying conditions differed between them (like the etiology of the proteinuric state) [72].

### 4.3. Pentoxifylline

Both insulin resistance and diabetes are linked to inflammation. This fact has generated a growing interest in anti-inflammatory therapies to slow diabetes and DKD progression [73]. Indeed, diabetes is now considered an inflammatory disease.

Pentoxifylline is a methylxanthine derivative and a nonspecific phosphodiesterase inhibitor of tumor necrosis factor (TNF-*α*) that has demonstrated an antiproteinuric effect in DKD [71, 74]. However, the heterogeneity and short follow-up of published studies have turned pentoxifylline away from the usual therapeutic arsenal against diabetes.

A well-designed long-term trial by Navarro-González et al. has been recently published. One hundred and sixty-nine diabetic patients with 3-4 stage CKD were randomized to placebo or pentoxifylline 600 mg daily one month, followed by 600 mg twice daily for 23 more months. All of them were receiving renin-angiotensin system blockers and the median of urinary albumin excretion was 1.1 grams per day. The study concludes that pentoxifylline slows renal disease progression (GFR slope) after the first year of treatment and maintained a statistically significant difference with placebo after 24 months [75]. Our group had previously published a small trial including 91 CKD patients, showing that pentoxifylline stabilizes renal function at 12 months, while patients in the placebo arm experienced a decline in renal function (estimated by MDRD) [76]. In the PREDIAN trial, urinary albumin excretion was reduced (mean of reduction difference of 20.6% between groups) in the pentoxifylline group at 6, 12, 18, and 24 months. Surrogate markers of inflammation also decreased at the end of the study in those patients receiving pentoxifylline [73]. These results therefore place pentoxifylline as one of the first-line drugs to be used in addition to renin-angiotensin system to avoid or at least decrease residual proteinuria in diabetic kidney disease.

### 4.4. Other Approaches

Some studies have tried to show beneficial effects of other drugs such as statins, aspirin, or rapamycin [77–80]. These anecdotal results should be cautiously managed, until studies designed with hard endpoints reveal further evidence.

## 5. Present and Future: Novel Drugs for Novel Approaches

An increasing knowledge of pathogenic mechanisms in DKD beyond proteinuria has enhanced studies of new molecules that could interfere in CKD progression (Table 2).

### 5.1. Endothelin Receptor Antagonists

Endothelins are small vasoactive peptides that influence hypertension and CKD through various mechanisms, including endothelial dysfunction, vasoconstriction, cell damage, and albuminuria [81, 82]. Their action is mediated through two families of receptors: endothelin-1 receptor (ETA) has been implied in the deleterious effects of endothelins, while endothelin-B receptors (ETB) act in the proximal tubule enhancing sodium excretion. All endothelin inhibitors have demonstrated positive effects on the kidney, by reducing proteinuria and renal function loss. However, the effect of inhibiting ETB results in inappropriate sodium retention, with more episodes of peripheral edema, congestive heart failure, and cardiovascular events. Unfortunately, this mishap happens with all known endothelin inhibitors, since they all have an effect on both ETA and ETB. Although first described with the earlier* bosentan*, molecules with higher selectivity on ETA over ETB like* sitaxsentan* and* avosentan* also showed these adverse events, which led to early termination of several trials [83–85]. Currently, the SONAR study is evaluating the effect of* atrasentan* on renal endpoints in type-2 diabetic patients [86], but it excludes patients with a history of peripheral edema or heart insufficiency and those with higher levels of type-B natriuretic peptide, so a limitation in its future indications is expected.

### 5.2. Antioxidant Therapies

Oxidative stress is part of the hyperglycemic-generated renal dysfunction. Several vitamin analogs and other molecules that inhibit redox reactions (such as* taurine*,* luteolin*,* D-saccharic 1,4-lactone*,* silybin,* or* hemin*) have proved to diminish kidney damage in animal models by normalizing superoxide dismutase, inducing hemoxygenase, or inhibiting NADPH oxidase [87–91].* N-Acetylcysteine*, which has been tested in many clinical trials for the prevention of contrast-induced nephropathy with controversial results [92], has not yet been proved effective in DKD, although studies are too small to be conclusive [93, 94].* Probucol* is another antioxidant drug that has shown nephroprotective capacity besides its hypolipidemic use [95].

Results regarding inhibition of xanthine oxidase are more promising.* Allopurinol* has already shown efficacy in preventing vascular events and slowing kidney function loss in several clinical trials [96, 97], some of which included diabetic patients. The ongoing clinical trials PEARL and FEATHER are currently investigating the specific usefulness of allopurinol, and its novel analogue* febuxostat*, in type 2 DKD [98, 99].

### 5.3. Transcription Factor Modulation

There are many attempts to interfere with the inflammatory pathways of DKD, aiming to interrupt the fibrotic pathogenesis. Some of these attempts are addressed at the earlier phases of the process, by inhibiting several transcription factors.

Protein-kinase activity is directly related to fibrosis [100], and several molecules have been studied to inhibit them.* Ruboxistaurin*, a protein-kinase C inhibitor, showed promising initial results in the fields of retinopathy and peripheral neuropathy [101, 102]. Data on DKD are very scarce with either negative effects [103] or a discreet benefit on protein excretion and GFR loss in the long term [104, 105]. However, these results have not been confirmed in larger populations or in patients with a decreased GFR. Other protein-kinase inhibitors are under current evaluation after associating renal benefits in animal models: tyrosine-kinase inhibitors* imatinib*,* nilotinib*,* genistein,* and* PP2* [106–109], Rho-kinase inhibitors* fasudil* and* Y27632* [110–112], p38-MAPK inhibitor* FR167653* [113, 114], phosphoinositide 3-kinase (PI3K) inhibitors* wortmannin*,* IC87114,* and* AS101* [115–117], or activin-like kinase 3 agonists (Alk-3)* THR-123* [118].

The Janus kinase-signal transducer and activator of transcription (JAK-STAT) system has also been related to kidney damage [119].* Baricitinib* is a JAK inhibitor currently under evaluation or rheumatoid arthritis that is also being studied for DKD [120].

Transcription processes can also be indirectly regulated through neurohormonal paths. The most studied pathway in this area is vitamin D receptor activation, but other ways are under evaluation. On the one hand, dopamine has been involved in blood flow regulation and hyperfiltration in earlier diabetic kidney disease. Experimental antagonism of D3 receptor with* D3-RA* showed beneficial effects on albuminuria and glomerulosclerosis [121], but results in humans are not yet available. On the other hand, serotonin has also been studied, and 5-hydroxytryptamine receptor antagonist* sarpogrelate*, which is more known for its antiplatelet action, has also demonstrated renal anti-inflammatory and antiproteinuric effects [122, 123] and is undergoing a phase IV randomized control trial. Melanocortin receptor activation has been evaluated in several nondiabetic proteinuric glomerulopathies, and treatment with subcutaneous* ACTH* has also shown efficacy in reducing proteinuria in DKD [124].

Finally, reinforcement of endogenous mechanisms that are inherently protective against hyperglycemia-derived kidney damage has also been tried. For example, exogenous administration of the adipocytokine* apelin* [125, 126] or of* activated protein C* [127] has renoprotective effects in DKD animals models. The exogenous activation of cannabinoid receptors has shown similar results [128, 129].

### 5.4. Antifibrotic and Anti-Inflammatory Agents

More downstream regulation of inflammatory and fibrosis cascades is also being explored. Treatments that inhibit cell adhesion and accumulation and cytokine production appear promising. In fact, TNF-*α* inhibition with* infliximab* or* etanercept* has been shown to decrease albuminuria and slow CKD progression in animal models, but further investigation in humans is required [130–132].

Transforming growth factor beta (TGF-*β*) blockade has been achieved through* pirfenidone*, currently approved for lung and liver fibrosis. Pilot studies showed a renal benefit but were halted due to adverse effects [133].* Tranilast*, currently approved for allergic states and keloids, showed a reduction in albuminuria in a small pilot study with diabetic patients [134, 135], but never underwent a larger clinical trial. After promising experimental data, there are several ongoing studies to evaluate the efficacy of specific anti-TGF-*β* monoclonal antibodies, such as* fresolimumab*, in various proteinuric nephropathies [136, 137]. Other TGF-*β* blockers have been tested in animal models but have not yet arrived to human subjects [138–140].

Connective tissue growth factor (CTGF) has also been implied in the process of renal fibrosis in DKD.* FG3019* is an anti-CTFG monoclonal antibody that showed albuminuria reduction in DKD [141] but has not been further investigated for this indication.

Reduction of chemokine production is also a potential treatment target in DKD. Several antagonists of the receptors CCR2 and CCR2/5 of the MCP-1 pathway (such as* CCX 140-B*,* TLK-19705*,* RS102895*,* PF-04634817,* or* BMS-813160*) have shown positive experimental results and some of them are being evaluated in clinical trials [142–145].

Another important family of proteins is that of matrix metalloproteinases (MMPs), mainly involved in extracellular matrix regulation [146]. Molecules with capacity to inhibit MMPs, such as the antibiotic agents* doxycycline* and* minocycline* or the newer* XL081* and* XL874*, were expected to have renoprotective effect, but when tested in humans, the impact was limited in magnitude and duration [147, 148]. Some trials are still under way.

Finally, one of the newest therapeutic approaches is based on developing molecules that target microRNA (miRNA) pathways [149]. These small noncoding RNA fragments are involved in gene expression regulation, and many of them have been identified with both protective and pathogenic roles [150]. Growing knowledge in their functions triggers interest in developing new drugs to either silence pathogenic miRNAs (via anti-miRNA oligonucleotides or similar agents) or to enhance renoprotective miRNAs (with mimics, vectors, or exosomes). To date, only one oligonucleotide has been tested in diabetic mice to prove its renoprotective efficacy [151].

### 5.5. Other Agents

Hyperglycemia-derived advanced glycation end-products (AGE) are known to have a pathogenic effect through the activation of their receptor (RAGE), causing protein dysfunction and altered collagen turnover activating metalloproteinases [152, 153]. Inhibition of RAGE with neutralizing antibodies reversed these pathogenic effects [154]. Several molecules that inhibit AGE formation, such as* pimagedine* or* pyridoxamine*, showed beneficial effects on animal models [155] but negative results or unacceptable adverse events in human trials [156–158].

Several oral adsorbents for uremic toxins have been tested, based on the hypothesis that reducing intestinal absorption of some of these toxins would diminish systemic inflammation and immune system activation. The most studied compound has been* AST-120*, also called* kremezin*, a spherical carbon preparation [159]. Initial studies cast hopeful results in early CKD [160, 161], but randomized clinical trials in moderate-to-severe CKD showed no effect [162]. A recent meta-analysis that included both kremezin and other adsorbents from Asian origin like* Ai Xi Te* and* Niaoduqing* granted a possible benefit in slowing the speed of renal loss, but without clear evidence [163].

Finally, other approaches are still in earlier stages of investigation. These include infusion of endovenous mesenchymal precursor cells or modulation of immune response through regulatory T cells or autophagy [164–166].

Many other attempts have revealed unsuccessful, despite arriving to phase II or III, clinical trials. This has been the case of* palosuran*, a urotensin-II receptor antagonist [167], or* sulodexide*, a glycosaminoglycan with anti-inflammatory properties in animal models [168–170].

## Figures and Tables

**Table 1:** Most relevant clinical trials assessing dual blockade of renin-angiotensin-aldosterone system in diabetic nephropathy.

Study	Patients and treatment arms	Commentary
Dual blockade using ACEI and ARB		

VA NEPHRON-D [41]	724 (losartan 100 mg/day)	Stopped due to adverse effects.
	724 (losartan 100 mg/day + lisinopril 10–40 mg/day)	Primary endpoint included change in eGFR, death, or end-stage renal disease.

ONTARGET [43]	8576 (ramipril 10 mg/day) 8542 (telmisartan 80 mg/day) 8502 (both)	Telmisartan equivalent to ramipril. No benefit of combination in proteinuria. Worse eGFR in combination group.

PRONEDI [42]	35 (lisinopril 40 mg/day) 28 (irbesartan 600 mg/day) 70 (lisinopril 20 mg/day + irbesartan 300 mg/day)	No benefit of combination in proteinuria or renal function.

Dual blockade using aliskiren		

ALTITUDE [45]	8561 (ACE/ARB + aliskiren 300 mg/day)	Stopped due to adverse effects. Greater reduction in proteinuria. Renal function was included in the primary endpoint.

AVOID [46]	298 (losartan 100 mg/day)	Greater reduction in proteinuria without differences in the decline of eGFR.
	301 (losartan 100 mg/day + aliskiren 150–300 mg/day)	No increased risk of adverse events.

Dual blockade using aldosterone blockers		

Sato et al. [47]	55 (spironolactone 25 mg/day to those patients with aldosterone escape after ACEI)	Early stage of CKD (eGFR >60 mL/min/1.73 m^2^). Greater reduction in proteinuria. No increased risk of adverse events.

Esteghamati et al. [48]	62 (enalapril 30–40 mg/day + losartan 50–100 mg/day)	Greater reduction in proteinuria.
	74 (spironolactone 25 mg/day + losartan 50 mg/day)	Greater loss of eGFR.

Epstein et al. [49]	91 (enalapril 20 mg/day) 91 (enalapril 20 mg/day + eplerenone 50 mg) 86 (enalapril 20 mg/day + eplerenone 100 mg/day)	Greater reduction in proteinuria in combination. No differences in eGFR reduction. No increased risk of hyperkalemia in combination.

ACEI: angiotensin converting enzyme inhibitor; ARB: angiotensin II receptor blockers; mg: milligram; eGFR: estimated glomerular filtration rate; CKD: chronic kidney disease.

**Table 2:** Summary of main pathogenic pathways and agents under evaluation for diabetic nephropathy.

Mechanism	Agent	Situation
Endothelin-receptor antagonism		

	Avosentan Atrasentan	Stopped due to adverse events Ongoing RCT

Antioxidant agents		

Direct renal effect	N-Acetylcysteine Probucol	Inconclusive results Apparent positive results

Xanthine oxidase inhibition	Allopurinol Febuxostat	Ongoing RCT Ongoing RCT

Transcription factor modulation		

Protein kinase modulation	Ruboxistaurin Imatinib Fasudil	Stopped due to adverse events Animal models/other indications Animal models

JAK-STAT pathway inhibition	Baricitinib	Ongoing RCT

Neurohormonal modification	D3-RA Sarpogrelate ACTH	Animal models Ongoing RCT Ongoing RCT

Endogenous agents	Apelin Activated protein C	Animal models Animal models

Antifibrotic agents		

Anti-TNF*α*	Infliximab	Animal models/other indications

Anti-TGF*β*	Pirfenidone Fresolimumab	Stopped due to adverse events Ongoing RCT

Anti-CTGF	FG3019	Animal models

Chemokine inhibition	CCX 140-B and others	Ongoing RCT

MMP inhibition	Tetracyclines XL081, XL874	Ongoing RCT Limited efficacy

miRNA modulation	LNA-anti-miR-192	Animal models

Other agents		

RAGE inhibition	Pimagedine Pyridoxamine	Stopped due to adverse events Ineffective

Oral adsorbents	Kremezin	Moderate efficacy

Urotensin-II inhibition	Palosuran	Ineffective

Glycosaminoglycans	Sulodexide	Ineffective

RCT: randomized controlled trial; JAK-STAT: Janus kinase-signal transducer and activator of transcription; ACTH: adrenocorticotropic hormone; TNF-*α*: tumor necrosis factor *α*; TGF-*β*, transforming growth factor *β*; CTG: connective tissue growth factor; miRNA: microRNA; RAGE: receptor of advance glycation end-products.
